# A novel machine learning approach generates personalized estimates of graft and patient survival to better inform older kidney transplant candidates

**DOI:** 10.3389/fimmu.2026.1881830

**Published:** 2026-07-08

**Authors:** Pierre-Luc Boivin, Jonathan Jalbert, Lévis Thériault, Yue Qi, Anastasiya Olek-Basanets, Héloise Cardinal

**Affiliations:** 1 Department of Mathematical and Industrial Engineering, Polytechnique Montréal, Montreal, QC, Canada; 2 &CO Collaborateurs Créatifs, Montreal, QC, Canada; 3 Department of Computer and Software Engineering, Polytechnique Montréal, Montreal, QC, Canada; 4 Centre d’Intégration et d’Analyse en Données Médicales, Centre de recherche du Centre hospitalier de l’Université de Montréal, Montreal, QC, Canada; 5 Immunopathology Axis, Centre de recherche du Centre hospitalier de l’Université de Montréal, Montreal, QC, Canada; 6 Department of Internal Medicine (Nephrology), Centre hospitalier de l’Université de Montréal, Montreal, QC, Canada

**Keywords:** calibration, expected post-transplant survival score, kidney donor risk index, kidney graft survival, kidney offer with lower potential longevity, machine learning approach, patient survival, random survival forest

## Abstract

**Introduction:**

An information most relevant to patients when deciding to accept a kidney offer is an estimate of its potential longevity. Using a novel machine learning approach, our aim was to develop a model that can output personalized curves estimating kidney graft and patient longevity to support clinical decision-making.

**Methods:**

We performed a retrospective cohort study in recipients of a first deceased donor kidney transplant aged ≥60 years between 2000 and 2020, using the United Network for Organ Sharing dataset. Outcomes were overall graft survival and patient survival. Independent variables included the Kidney Donor Risk Index (KDRI) and recipient-related variables. Random survival forest models were trained on 70% and validated on 30% of the dataset.

**Results:**

The study cohort included 57, 280 patients, amongst whom 27, 244 (48%) experienced graft loss, while 25, 210 (44%) died during a median follow-up of 4.3 years. The most important variables in tree development for graft survival were recipient age, recipient diabetes, KDRI, recipient ethnicity, and time on dialysis. For patient survival, these variables were recipient age, recipient diabetes, time on dialysis, recipient cause of chronic kidney disease, and recipient HCV status. The difference in expected graft survival between low and high KDRI kidneys decreased when recipient characteristics, in particular age, were accounted for. KDRI had a negligible impact on mortality.

**Discussion:**

While implementation studies are needed, the individualized curves provided by our novel machine learning approach have the potential to support shared decision-making to a greater extent than the KDRI alone.

## Introduction

Kidney transplantation is the best therapeutic option for patients suffering from end-stage kidney disease (ESKD), improving both quality of life and life expectancy when compared to remaining on dialysis (1, 2). The gap between the need and the availability of kidneys for transplantation has not diminished significantly in the past decade (3), leading to thousands of patients waiting for a kidney in North America (3, 4). To fill this gap, using kidneys from donors who have risk features for lower longevity can be a beneficial alternative to dialysis for many patients (5–8). This is especially relevant for older transplant candidates (7) who now represent over 25% of kidney transplant candidates, an ever growing proportion (9). Nevertheless, the decision to accept or decline a kidney offer that has lower potential longevity must be made through a shared-decision making process between the transplant physician and the transplant candidate (10–12). To achieve this, relevant information must be provided to patients so that they can assess which option-accept or decline the offer- is most suited to their preferences and life-goals (10, 13).

At present time, the metric most often used to convey information to transplant candidates relative to the quality of a kidney offer is the Kidney Donor Profile Index (KDPI), a metric comprised between 0 and 100% that indicates the quality of a donor relative to all other donors of a reference year, with higher values indicating lower quality. The metric is based on a risk score, the Kidney Donor Risk Index (KDRI) that comprises donor-related variables and is associated with kidney graft longevity (14). The KDPI hence allows for a comparison between the quality of a current offer and the overall quality of kidneys in the donor pool, but does not directly estimate the longevity of the organ. Through focus group discussions with kidney transplant candidates and recipients, we and others (15, 16) have previously identified that the most relevant information to convey to patients in order for them to participate to the decision to accept or decline an offer is an estimate of graft longevity. In a recent qualitative study involving older patients suffering from ESKD, an expected graft survival of 3–5 years was deemed sufficient to accept a kidney offer with potential lower longevity (16). Providing estimates of patient and graft longevity is often difficult as donor, recipient and matching (HLA, size, age) characteristics between the donor and the recipient must be considered. Here, we propose a machine learning tree-based approach to provide personalized, well-calibrated kidney graft and patient survival curves. The reasons for choosing a machine learning based approach rather than a standard Cox model are that these approaches are better at handling non-linearities and interactions between variables and can directly generate survival curves. Those personalized survival curves could help transplant physicians convey relevant information on expected graft longevity to transplant candidates during education sessions while on the wait list or at the time of a kidney offer, to enhance shared decision-making.

## Materials and methods

### Patients and setting

We performed a retrospective cohort study in consecutive patients registered in the United Network for Organ Sharing database who received a first deceased donor single kidney transplant between January 1^st^, 2000 and January 1^st^, 2020. Because kidneys from donors with characteristics associated with lower expected longevity (extended criteria donors or high KDPI donors) are usually not offered to young candidates, we excluded patients who were less than 60 at the time of transplant. We also excluded those who received ABO incompatible transplantations, those who had missing or invalid donor height (<30 or >250 cm), weight (<10 or >400kg), or terminal serum creatinine (<0.2 or > 23 mg/dl), missing dates important in outcome definitions, recipients of kidneys that were donated after uncontrolled cardiocirculatory arrest or originating from donors less than 5 years of age. All patients were followed until graft loss (return to dialysis or retransplantation), patient death, or June 30^th^, 2021, whichever occurred first. If no graft loss or mortality events were noted during the study period, follow-up was administratively censored at the time of the last report of the transplant center to UNOS for the graft loss outcome, as reliable information for this outcome could not be obtained afterwards. There was no administrative censorship for mortality events at the time of the center’s last report to UNOS, since death dates are verified in UNOS through national registries. The project was approved by the Centre hospitalier de l’Université de Montréal ethics review board (MP-02-2017-6870, CE 16.188 – CA).

### Outcomes

The primary outcome was overall graft survival, defined as the time elapsed between the transplant date and return to dialysis, retransplantation or patient death. The secondary outcome was patient survival, defined as time elapsed between transplantation and death.

### Independent variables

Independent variables included donor and recipient related characteristics. The following donor characteristics were included: 10-variable KDRI (score includes donor age, ethnicity, height, weight, type (neurologically deceased versus after cardiocirculatory arrest), cause of death, history of hypertension, diabetes, seropositivity for hepatitis C, terminal serum creatinine), donor sex, history of cigarette smoking, and cocaine use. Recipient-related variables were: recipient age, sex, ethnicity, cause of ESKD, diabetes, peripheral vascular disease, hepatitis C seropositivity, hepatitis B surface antigen (HbsAg) positivity, history of malignancy, height, body mass index, time on dialysis prior to transplantation, pre-transplant panel reactive antibodies. Donor-recipient matching-related variables were: A, B and DR mismatches, cytomegalovirus (CMV) serology and Epstein-Barr virus (EBV) serology mismatches.

### Model training

To generate personalized graft and patient survival curves, we developed a model inspired by decision trees but adapted for survival prediction. First, the full cohort was randomly divided into a training set (70% of observations) and a test set (30% of observations). Using the training set, the algorithm identified the independent variable and the cut-off threshold that split the set into two subgroups, maximizing heterogeneity between them, as is commonly performed in regression trees. However, we adopted a novel approach for the splitting measure: we chose the split that maximized the distance between the two Kaplan–Meier survival curves of the subgroups. This distance was measured using the Kolmogorov–Smirnov test statistic, which corresponds to the maximal vertical distance between the two curves. To identify the variables that should represent nodes and relevant thresholds for split (in the case of continuous variables), the algorithm tested every binary combination of categorical variables and every possible split at various increments (continuous variables), selecting the split that maximized the Kolmogorov–Smirnov statistic. This process was then repeated recursively for each subgroup, and iterations continued until one of the stopping criteria was met. The stopping criteria were: (1) the number of events in a subgroup fell below a minimum threshold required to produce smooth Kaplan–Meier curves, or (2) a maximum tree depth was reached.

To improve the robustness of the predictions, we then created a random forest of survival trees (RSF). The RSF consisted of multiple trees, each built after shuffling the observations and excluding a different 20% subset of the training data. For prediction, donor and recipient characteristics are passed through every tree in the forest. The individuals in each terminal node are then aggregated to generate personalized patient or graft survival curves.

### Model calibration

For the purposes of this study, the most important property to evaluate is the model calibration as our aim is to provide trustworthy, well-calibrated information to transplant physicians and candidates to help them choose to accept or decline an offer. Hence, we want to ensure that 1) the individual survival distributions we provide to patients can be trusted, *i.e.*, that the predicted values are consistent with the observed ones (17); 2) the models improve predictive ability compared to a naïve approach (Kaplan-Meier curves for graft and patient survival for the full cohort) or scores that are currently used in the clinical setting (KDRI/KDPI for graft survival and Expected Post-Transplant Survival (EPTS) score for patient survival). Calibration was evaluated on the test set (30% of observations never seen by the model during training). First, we performed a D-calibration visual assessment (18) where patients are classified in bins corresponding to the deciles of their *predicted* probability of the event at the time they actually experienced the event. For right-censored follow-up observations, each patient is assigned to bins of lower deciles than the predicted probability at the time of censorship, and an even fraction of the patient’s presence is added to each of those bins. If the model is well-calibrated, approximately 10% of all patients should be located in each decile of the predicted probability of the event. Large p-values using this approach suggest that there is no evidence that observed distribution differs from the expected uniform distribution and that the model is well-calibrated. Second, we examined whether the Integrated Brier Score (IBS) was significantly lower using our models than with other approaches (19, 20). To achieve this, we generated 1000 bootstrap samples from the test dataset. For overall graft survival, the IBS of the Kaplan-Meier estimator, KDRI, and RSF models was evaluated on each bootstrap sample. For patient survival, the Kaplan-Meier estimator, EPTS, and RSF models were assessed. We then calculated the differences in IBS between KDRI and RSF for overall graft survival, and between EPTS and RSF for patient survival. The mean differences and corresponding 95% confidence intervals were estimated using the bootstrap samples. To evaluated the relative importance of individual features, or independent variables, in outcome prediction, we conducted a permutation feature importance analysis. This approach involves randomly permuting the values of each feature and measuring the resulting degradation in the D-calibration test statistic. By disrupting the relationship between the selected feature and the outcome, the analysis quantifies the extent to which model performance depends on each feature. The permutation procedure was repeated 10 times for each feature, and the mean importance score along with its standard error were reported. Last, we performed a grid search to define the hyperparameters (number of trees in the forest, maximal depth of the trees, minimal number of patients with events in the terminal subgroups, resolution of the increments in continuous variables where data splits occur) that yielded the best calibration possible, as measured by the chi-square statistic. For categorical variables, missing data were categorized as a separate category, whereas mean values were imputed for missing continuous variables.

## Results

**Figure 1:**
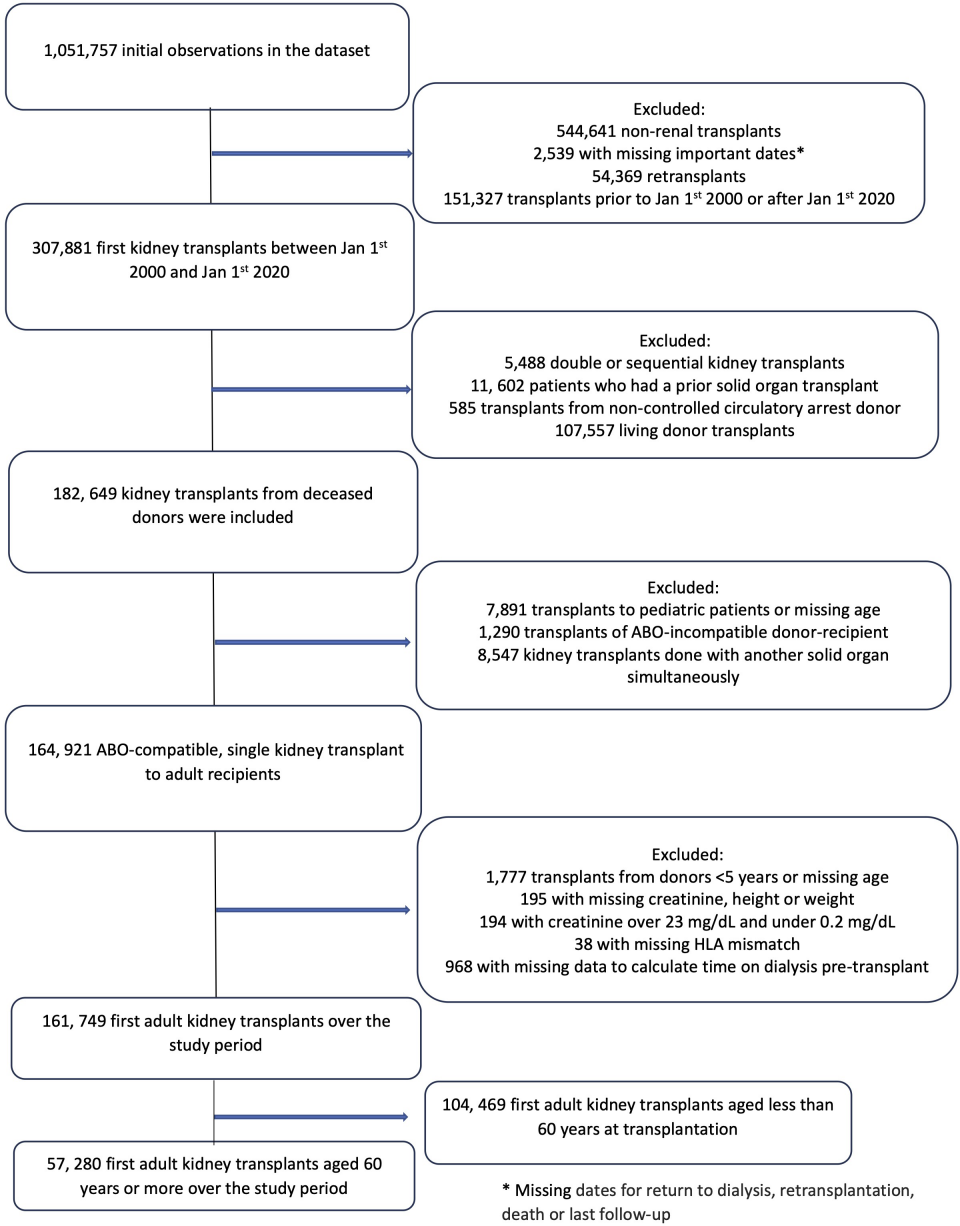
Patient flow chart. There is no legend for this image.

After applying the exclusion criteria described above, the study cohort included 57, 280 first deceased donor older kidney transplant recipients (**Figure 1**). Amongst the latter, 27, 244 (48%) experienced the primary outcome of return to dialysis (n=4,706), retransplantation (n=282) or death (n=22, 256) over a median of years of follow-up of 4.3 years (interquartile range (IQR) 2.0 to 7.5 years). During the study period, 25, 210 patients (44%) died. The donor and recipient characteristics can be found in **Table 1** along with the frequency of missing data.

**Table 1:** Donor and recipient characteristics at the time of transplantation.

Recipient characteristics (n=57, 280)	
Mean age at transplantation in years (standard deviation, SD)	66 (5)
Median time in dialysis in years, (interquartile range (IQR))	3.3 (1.6, 5.3)
Mean BMI in kg/m^2^, (SD)	28 (5)
Median pre-transplant panel reactive antibodies, (IQR)	0 (0-5)
Male sex, n (%)	35, 132 (61)
Positive HCV, n (%)	2, 954 (5)
Positive HbsAg, n (%) *(missing data=2, 489)*	962 (2)
Donor (D)-recipient (R) CMV serology, n (%) *(missing data=1, 842) * D+/R- D+/R+ or D-/R+ D-/R-	8, 944 (16) 40, 488 (73) 6, 006 (11)
Diabetes, n (%) *(missing data n=33)*	28, 541 (50)
Cause of chronic kidney disease, n (%) Glomerular diseases Hypertension/vascular Diabetes Autoimmune Polycystic and other cystic kidney disease Other or Unknown	6, 358 (11) 15, 764 (28) 23, 298 (41) 1, 144 (2) 4, 803 (8) 5, 813 (10)
Ethnic origin, n (%) Caucasian African American Hispanic Asian Other or unknown	28, 840 (50) 15, 514 (27) 7, 781 (14) 4, 001 (7) 1, 144 (2)
Peripheral vascular disease, n (%) *(missing data n=1, 377)*	5, 745 (10)
Previous malignancy, n (%) *(missing data n=2, 193)*	6, 420 (12)
Donor (D)-recipient (R) EBV serology, n (%) Positive EBV mismatch (D +/R-) Negative EBV mismatch (R+ or D-) Undetermined EBV mismatch	3, 457 (6) 42, 864 (75) 10, 959 (19)
HLA A-B-DR mismatches, n (%) 0 1 2 3 4 5 6	4, 115 (7) 1, 169 (2) 2,502 (4) 7, 364 (13) 14, 811 (26) 18, 101 (32) 9, 218 (16)
Donor characteristics	
Mean age in years, (SD)	44 (15)
Mean height in cm, (SD)	171 (12)
Mean weight in kg, (SD)	82 (22)
Median creatinine in mg/dL, (IQR)	1.0 (0.7-1.4)
Mean Kidney Donor Risk Index*, (SD)	1.37 (0.4)
Male donor sex, n (%)	32, 950 (58)
Stroke as cause of death, n (%)	22, 800 (40)
Donor after cardiocirculatory arrest, n (%)	9, 276 (16)
Positive HCV serology, n (%)	2, 519 (4)
Smoking history, n (%) *(missing data n=906) * Active smoking Past smoking Never smoker	13, 710 (24) 3, 078 (5) 39, 586 (70)
Positive history of cocaine use (active or prior), n (%) *(missing data n=981)*	9, 497 (17)
Ethnic origin, n (%) Caucasian African American Hispanic Asian Other or unknown	40, 941 (71) 7, 174 (13) 6, 843 (12) 1, 439 (3) 883 (2)
Hypertension, n (%) *(missing data n=442)*	21, 044 (37)
Diabetes, n (%) *(missing data n=357)*	5, 470 (10)

*As per the original equation (14).

### Random forest model for kidney graft and patient graft survival curves

**Figure 2:**
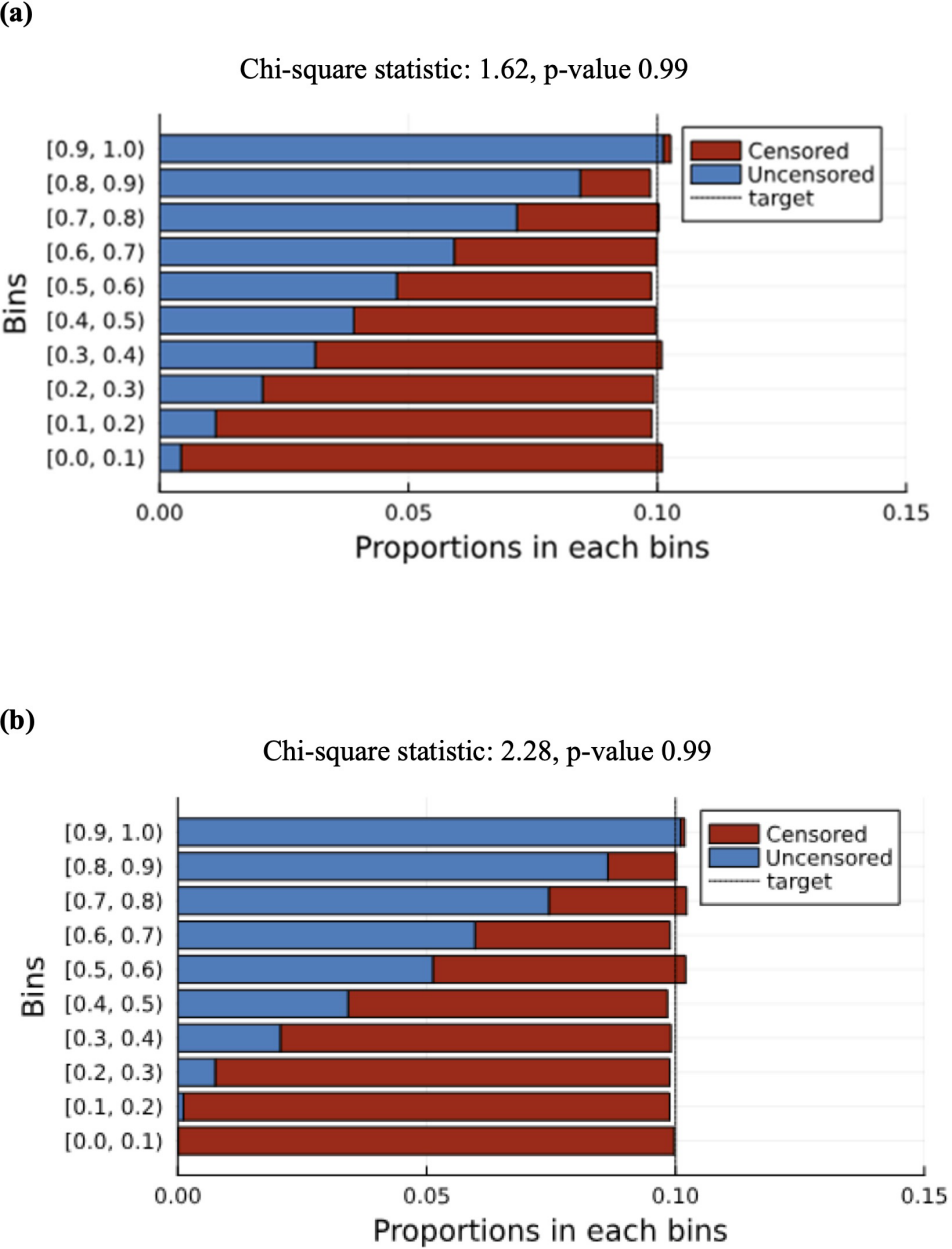
Calibration evaluation for graft and patient survival models. Distribution-calibration (D-calibration) assessment for the overall graft survival model **(A)** and the patient survival model **(B)**. To evaluate D-calibration, patients in the test set are assigned to bins that represent the deciles of the predicted probability of the event of interest at the time they actually experienced the event of interest. The method consists of calculating the predicted probabilities of the events at their true respective time of events for all patients in the test set. These probabilities will be sorted in ascending order and separated in deciles (bins), from the lowest to the highest probabilities. Hence, if his predicted probability was in the second decile of predicted probabilities of the event at 5 years (for instance 16%), he will be assigned to the 0.1-0.2 bin. For right-censored follow-up observations (no event of interest during follow-up), an even fraction of each patient in the test set is assigned to bins with lower probabilities than the predicted probability of the event for that patient at the time of censorship. For instance, if a patient experiences no event during a 6-year follow-up and is right-censored at that time, and his predicted probability for the event at 6 years is in the third decile (0.2-0.3 bin), then ‘0.5 patient’ will be assigned to the 0-0.1 bin and ‘0.5 patient’ will be assigned to the 0.1-0.2 bin. In a well-calibrated model, 10% of all patients should be located in each decile of the predicted probability of the event. Large p-values using this approach suggest that there is no evidence that observed distribution differs from the expected uniform distribution and that the model is well-calibrated.

**Figure 3:**
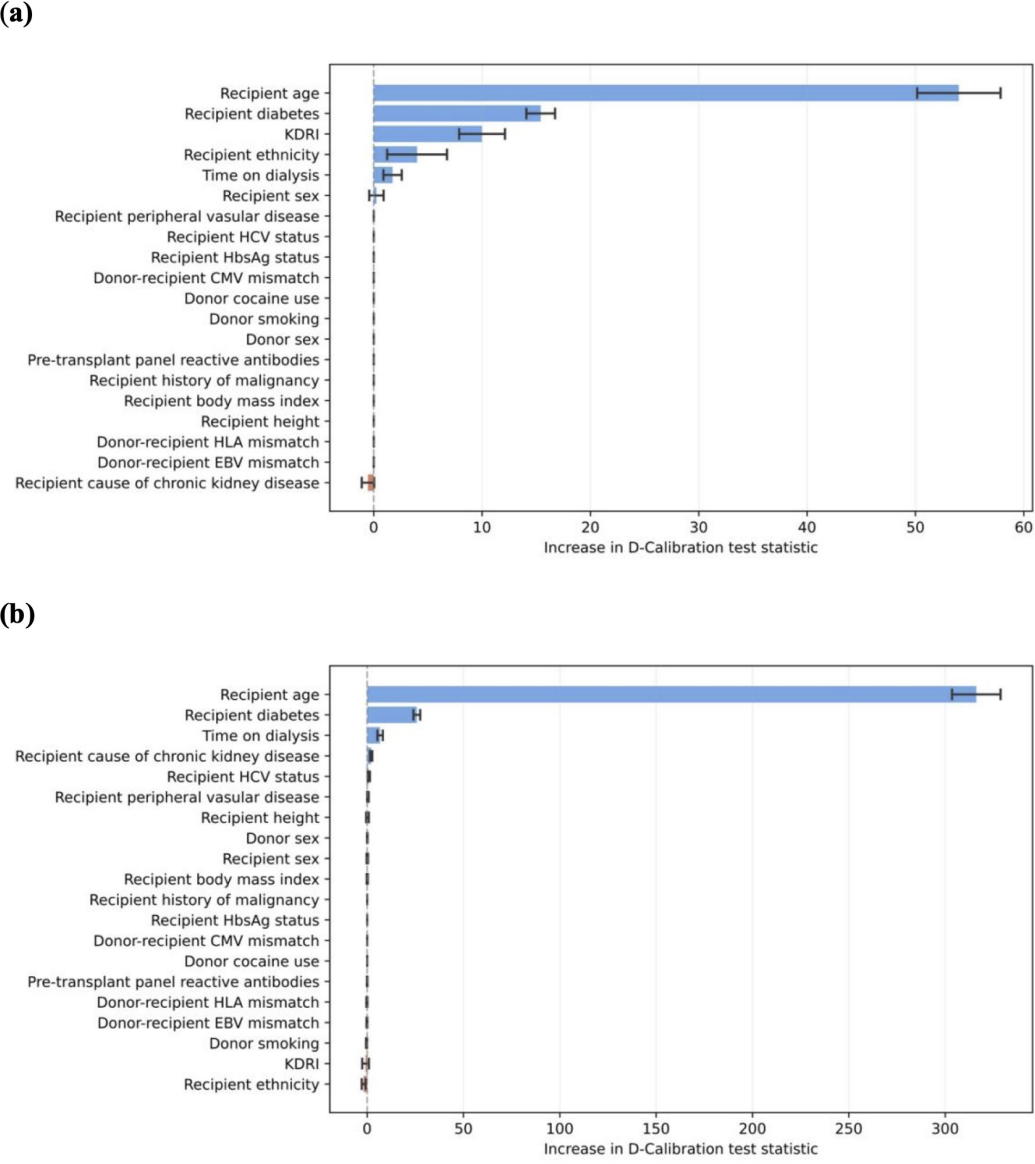
Permutation feature importance for the Random Survival Forest models predicting overall graft survival and patient survival. Feature importance was estimated using 10 permutation repetitions per feature. **(A)** Permutation feature importance for the Random Survival Forest model predicting overall graft survival. **(B)** Permutation feature importance for the Random Survival Forest model predicting patient survival.

We built 2 different models, aimed to provide to transplant physicians and candidates personalized estimates of overall graft and patient survival. After the grid search was complete, the hyperparameters that yielded the optimal calibration performance for the models were obtained and are shown in **Table 2**. All models were well calibrated (**Figure 2**). The Brier scores for our random forest models were significantly lower than those observed for a naïve Kaplan-Meier approach, the KDRI and the EPTS, as the 95% confidence intervals for the differences were not overlapping the null value, indicating better calibration (**Table 3**). The permutation feature importance analysis indicated that the variables most important in tree development for graft survival were recipient age, recipient diabetes, KDRI, recipient ethnicity, and time on dialysis. For patient survival, the most influential variables were recipient age, recipient diabetes, time on dialysis, recipient cause of chronic kidney disease and recipient HCV status (**Figure 3**).

**Table 2:** Grid search results for the optimal hyperparameters for graft and patient survival models.

Model	Number of trees	Maximal depth	Minimal number of events in terminal leaf	Percentile increments used to create data splits for continuous variables
Overall graft survival	10	50	200	10
Patient survival	5	10	200	5

**Table 3:** Comparisons of integrated brier scores (IBS) for graft and patient survival between various models*.

Model	Kaplan-Meier	KDRI	EPTS	RSF	IBS difference
Overall graft survival	0.1916 ± 0.0013	0.1873 ± 0.0013	–	0.1806 ± 0.0013	0.0068 (0.0058, 0.0078)
Patient survival	0.1837 ± 0.0012	–	0.1755 ± 0.0012	0.1743 ± 0.0012	0.0012 (0.0005, 0.0018)

*IBS difference denotes the difference in IBS between KDRI and RSF for overall graft survival, and between EPTS and RSF for patient survival. Mean differences are presented with 95% confidence intervals in parentheses.

Values reported as *x* ± *y* represent the mean ± standard deviation (SD).

Abbreviations: RSF, Random Survival Forest; KDRI, Kidney Donor Risk Index; EPTS, Estimated Post-Transplant Survival score.

### Clinical case vignettes for the different models

To illustrate the clinical usefulness of our approach, we will compare the information provided with our random forest model (which includes the KDPI as one of the independent variables) *versus* the KDPI alone, which is widely used for providing information to patients (21) as well as for utility matching in the US. The KDPIs reported are relative to the distribution of all donors in 2023.

#### Providing personalized estimates of overall graft survival

We present the use cases of 4 randomly chosen transplant candidates aged 60, 65, 70 and 75 years who need to be informed of the expected duration of function of kidney offers he/she may receive: *Offer 1* has a KDRI of 0.87, the 10^th^ percentile of the KDRI distribution of the study cohort, equivalent to a KDPI of 37% for all 2023 donors; *Offer 2* has a KDRI of 1.96, the 90^th^ percentile of the KDRI distribution of the study cohort, equivalent to a KDPI of 98% for all 2023 donors. If the KDPI alone is used, then the candidates will all be informed that 37% of all offers have better characteristics (higher expected longevity) than *Offer 1*, but that 98% of all offers have better expected longevity than *Offer 2.* While this may provide an estimate of the quality of an offer relative to all others, it does not provide estimates of graft longevity *per se.*

**Figure 4:**
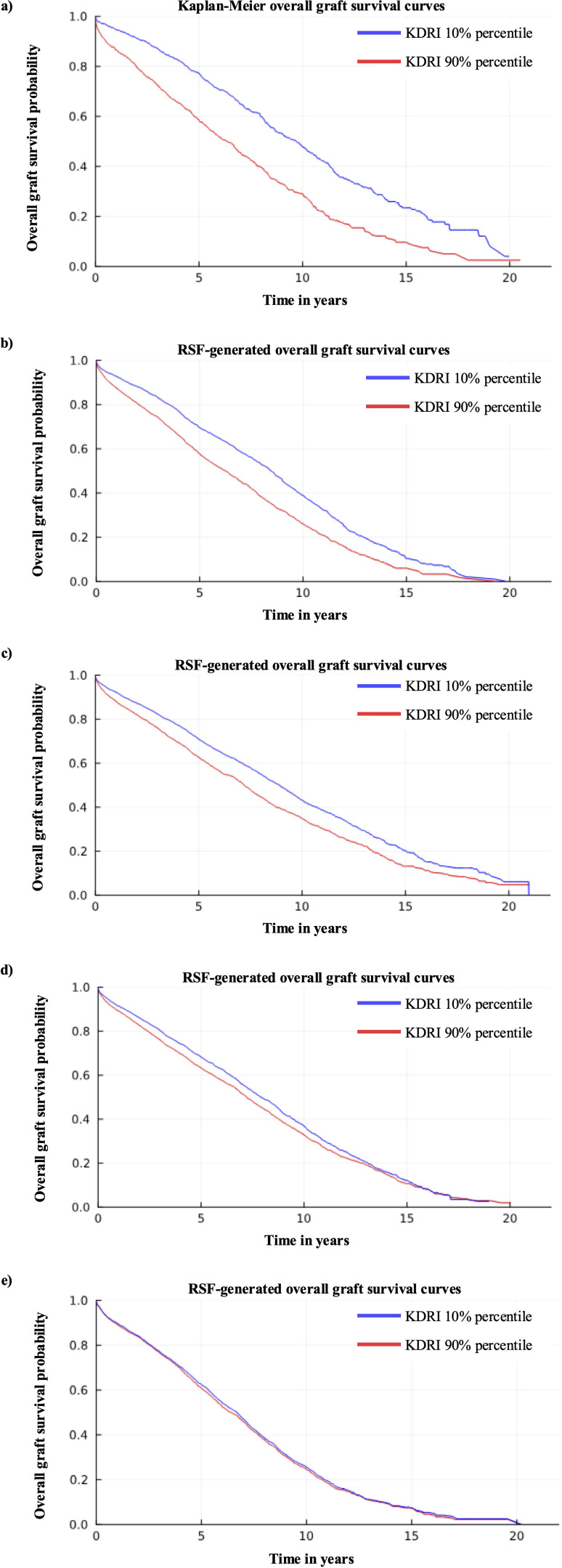
Overall graft survival curves using the Kidney Donor Risk Index (KDRI*) alone and the KDRI integrated in our personalized tree-based approach. **(A)** Overall kidney graft survival Kaplan-Meier curves for patients receiving kidneys from donors with KDRIs at the 10^th^ and 90^th^ percentile of the study cohort distribution. **(B)** RSF-based personalized overall graft survival curve generated for 1 randomly selected 60-year old kidney transplant recipient receiving either a kidney with a KDRI at the 10^th^ or at the 90^th^ percentile of the study cohort distribution. **(C)** RSF-based personalized overall graft survival curve generated for 1 randomly selected 65-year old kidney transplant recipient receiving either a kidney with a KDRI at the 10^th^ or at the 90^th^ percentile of the study cohort distribution. **(D)** RSF-based personalized overall graft survival curve generated for 1 randomly selected 70-year old kidney transplant recipient receiving either a kidney with a KDRI at the 10^th^ or at the 90^th^ percentile of the study cohort distribution. **(E)** RSF-based personalized overall graft survival curve generated for 1 randomly selected 75-year old kidney transplant recipient receiving either a kidney with a KDRI at the 10^th^ or at the 90^th^ percentile of the study cohort distribution. *KDRI located at the 10^th^ and 90^th^ percentile of the distribution in the current study cohort are equivalent to Kidney Donor Profile Index (KDPI) of 37% and 98% relative to all 2023 donors.

The KDPI alone can be used to provide overall graft survival estimates for these candidates. For instance, Kaplan-Meier graft survival curves by KDPI for *Offer 1* and *Offer 2* can be plotted and discussed with candidates (**Figure 4A**). These would indicate a 77% probability of being alive and off dialysis 5 years post transplant (*versus* 58% for *Offer 2)*, a figure which would drop to 48% 10 years post transplant (*versus* 28% for *Offer 2*). These differences are of large magnitude. When our approach is used, personalized curves that account for the KDPI, but also for other recipient and donor-recipient matching characteristics, are generated (**Figures 4B–E**). For our randomly chosen candidates aged 60–75 years, when the survival curves generated by our approach are compared to those using the KDPI alone, we observe that 1) the absolute differences in estimated overall graft survival between *Offer 1* and *Offer 2* are of decreased magnitude than when the KDPI alone is used. Using the KDPI only, the difference in expected 5-year graft survival is close to 20%, while these differences are ≤10% with our approach; 2) the absolute differences in overall graft survival between low and high KDPI kidneys diminish as recipient age increases; 3) For recipients of the same age (**Supplementary Figures 1**, **2**), the relative importance of KDPI in overall graft survival curves output can vary between recipients, indicating that multiple recipient (and non-KDPI donor-related) characteristics are taken into account to generate survival estimates.

#### Providing personalized estimates of patient survival

**Figure 5:**
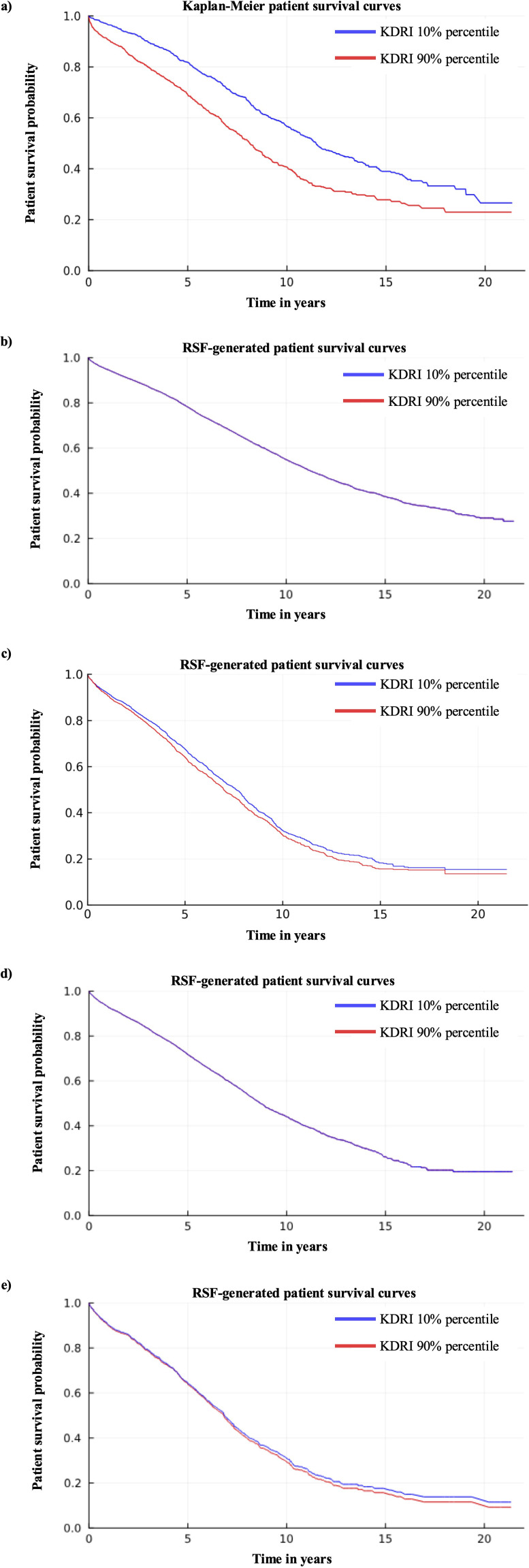
Patient survival curves using the Kidney Donor Risk Index (KDRI*) alone and the KDRI integrated in our personalized tree-based approach. **(A)** Patient survival Kaplan-Meier curves for patients receiving kidneys from donors with KDRIs at the 10^th^ and 90^th^ percentile of the study cohort distribution. **(B)** Random survival forest (RSF)-based personalized patient survival curve generated for 1 randomly selected 60-year old kidney transplant recipient receiving either a kidney with a KDRI at the 10^th^ or at the 90^th^ percentile of the study cohort distribution. **(C)** RSF-based personalized patient survival curve generated for 1 randomly selected 65-year old kidney transplant recipient receiving either a kidney with a KDRI at the 10^th^ or at the 90^th^ percentile of the study cohort distribution. **(D)** RSF-based personalized patient survival curve generated for 1 randomly selected 70-year old kidney transplant recipient receiving either a kidney with a KDRI at the 10^th^ or at the 90^th^ percentile of the study cohort distribution. **(E)** RSF-based personalized patient survival curve generated for 1 randomly selected 75-year old kidney transplant recipient receiving either a kidney with a KDRI at the 10^th^ or at the 90^th^ percentile of the study cohort distribution.

The KDRI was not developed to predict patient survival. However, physicians may want to inform patients of the impact of accepting a kidney that has lower expected longevity (a high KDPI) on their expected survival. In **Figure 5A**, patient survival curves are generated for *Offer 1* and *Offer 2* using only the KDPI. Absolute differences in patient survival of 10% 5 years post transplant and close to 20% at 10 years are observed between recipients of high and low KDPI kidneys. In contrast, using our approach to inform 4 randomly chosen patients aged 60–75 years, we can observe that when recipient characteristics are accounted for, there is no impact of accepting a high versus a low KDPI offer on patient survival (**Figures 5B–E**) and **Supplementary Figures 3**, **4**.

## Discussion

While accepting a high KDPI kidney is a better option in terms of survival than remaining on dialysis in a majority of cases (7), patients need and wish to be informed of the impact of that decision in comparison to accepting a standard donor (15, 16). Here, we provide a novel, well calibrated tool that 1) focuses on transplant outcomes-graft and patient survival- that are relevant to patients; 2) provides a visual display of personalized information to transplant candidates and physicians on the expected impact of receiving a kidney transplant from a donor with risk characteristics for lower graft longevity. We developed a tree-based, random survival forest model because in contrast to standard Cox regression survival analysis, machine learning techniques notably perform well in taking into account various interactions between independent variables in outcomes predictions, and do not presuppose that the association between independent variables and outcome are constant in time (22). Furthermore, Cox standard regression does not directly provide survival probabilities/curves and Kaplan-Meier curves yield population averages that are not personalized. Our proposed approach was successful in addressing these limitations. The individualized curves our approach outputs provide more precise information, showing potential for better supporting shared decision-making compared to using a KDPI number alone.

Donor characteristics, expressed as the KDRI, have previously been shown to interact with recipient characteristics (age, diabetes, ethnicity) in predicting graft survival (23). Although our random forest models did not include interaction testing, our findings are in line with the occurrence of such interactions between recipient and donor-recipient matching characteristics. For instance in the clinical vignette we provide using our novel approach, the difference in overall graft survival curves according to KDPI was modest when recipient characteristics were considered. This information is distinct from the survival curves generated using the KDPI alone. This is most likely due to selection bias, since the case mix of patients who receive high and low KDPI differs (*ie*, higher KDPI offers are directed to older candidates or those with a higher comorbidity burden). The absence of association between donor quality and elderly kidney transplant recipient survival has been reported by others (5, 24). although one study reported that elderly recipients of older deceased after cardiocirculatory arrest donors may experience a greater risk of 5-year mortality than if they had received a young neurologically deceased donor (5). We do not make that distinction as the death process (after cardiocirculatory arrest *versus* neurologically deceased) is included in the KDRI.

We expect that our novel approach, which provides a personalized graphical display of estimated graft and patient longevity, will prove useful in informing patients so that they can make the best decision regarding their priorities and life goals. For instance in the case presented, using the KDPI alone serves to compare the quality of an offer relative to the donor pool. It does not inform patients in a meaningful way on the impact of the donor quality on graft longevity. This could have an adverse impact on graft acceptance rates (25), as patients may perceive that higher KDPI offers will not provide them with sufficient graft longevity. When our graphical approach is used, on the other hand, patients can evaluate the probability of being alive and off dialysis at different time points post transplant according to the quality of the offer, as evaluated by the KDPI. With this information, they can assess whether high KDPI kidneys are expected to provide them with an acceptable probability of function in the future. This acceptable probability will vary by patients. In a recent study of older patients with ESKD, the majority of participants responded that an allograft functioning 3–5 years post transplant was worth taking the risk of accepting a kidney with a high KDPI (16). By visualizing the curves presented, patients will be able to judge whether the survival probabilities are sufficient at 3–5 years for them to accept offers with high KDPIs. In the cases we present using our approach, even the high KDPI kidneys provide survival estimates above 50% more than 5 years post transplant, the absolute differences between the high and low KDPI offers in terms of overall graft survival probability at 5 years are minor from a clinical stand-point and tend to diminish as recipient age increases, which is expected since death with function is more likely as recipients age (26). Even for recipients of the same age, the relative importance of KDRI in graft survival curves generated vary according to individual recipient characteristics. This observation is in line with our goal of providing personalized survival curves which take into account multiple patient and donor (KDRI-related or not) variables. Importantly, patients will be able to make their own opinion as to whether this difference in survival probability is meaningful, balancing this difference with longer time on dialysis should an offer be refused. This is especially relevant since for most patients aged 50 years and above, even the highest KDPI kidneys provide a survival benefit when compared to remaining on the wait list for transplantation _(_7_)._

Three recent studies (8, 27, 28) combine the KDRI with either EPTS or other recipient characteristics to predict post-transplant survival. In these studies, the main objective was to estimate patient survival (8, 27, 28) and/or kidney graft survival (28) depending on whether a patient accepted a kidney offer of variable quality, as estimated by the KDRI, or chose to remain on dialysis to wait for a better offer. Despite clear evidence of benefit from kidney transplantation in terms of patient survival-even with high KDRI kidneys-dating back to 2014 (7), kidneys labeled ‘high KDPI’ still have a high rate of discard (25). This risk adversity may in part be due to the scarcity of information on whether accepting a high KDRI kidney leads not only to improved patient survival but also prolonged time alive and off dialysis (overall graft survival). While Wey et al. (28) examine this issue, they report data only up to 3 years post transplant, a short-term endpoint. This is relevant since the impact of high KDRI on graft survival increases with time post transplant (21). Qualitative studies show that for most transplant recipients, being alive off dialysis is more important than surviving itself (29) and that remaining alive after kidney graft failure is associated with an important adverse impact on patient’s well-being (30). Hence, transplant patients and physicians need to be informed of the long-term impact of accepting donors of different quality on overall graft survival. Our approach is unique in this regard, providing a visual display of the long-term impact of accepting high KDRI kidneys on overall graft survival based on recipient/donor characteristics and being able to make survival predictions by changing only donor quality/KDRI while keeping other patient characteristics constant.

Our study has certain limitations. First, we did not perform validation on an external cohort, hence the current models are only calibrated for the US population aged 60 and over and are not directly applicable to recipients from other regions. Nevertheless, the code we developed is made public with the current publication and models can be retrained on local dataset so that random forests can be rebuilt and model parameters determined to yield the best calibration metrics possible. The accuracy of predictions in any given model is contingent on the quality of the data entry and the number of observations. Here, we used the UNOS registry to train and validate the model. While this data source has the advantage of comprising a large number of patients, registry data often lacks the granularity of center-level data that is accessible to transplant physicians. Hence, the approach proposed aims at supporting clinical and shared decision-making, and cannot replace clinical judgment. Immunosuppression can influence post transplant outcomes and its inclusion in the models could have enhanced the precision of survival estimates. However, the choice of immunosuppression can be informative of early graft outcomes, which can in turn affect patient and/or graft survival. As the post transplant evolution is unknown before or at the time of the offer, which is when the models are meant to be used, we chose not to include immunosuppression or any post transplant variable in the models. Last, this tool should be evaluated in real-world decision-making and communication studies before widespread implementation, as potential challenges to its use include poor health literacy, especially in older patients (31).

In conclusion, we have developed models that generate a personalized visual display of survival estimates that inform patients and physicians on the impact of the quality of an offer on post-transplant graft and patient longevity. This approach can be used at the time of organ offer, to compare the impact of the quality of a current offer on survival probabilities to that of other offers. It can also be used for patient education at the time of listing or during the waiting time to transplantation so that patients can pre-consent to receiving high KDPI offers and be prepared for shared decision-making at the time of an offer. Instead of focusing on ranking the quality of an offer relative to the donor pool when educating patients or at the time an offer is made, we suggest that providing personalized estimates of the expected longevity of offers to patients would best respond to their need for information. Further studies will be needed to evaluate how this new approach could be implemented and whether its use could eventually decrease the discard of high KDPI kidneys.

## Data Availability

The data analyzed in this study is subject to the following licenses/restrictions: Data comes from UNOS and access procedures are in public domain. Requests to access these datasets should be directed to UNOS website.

## Supplementary material

Supplementary Figure 1 Random survival forest-generated overall graft survival estimates for 4 randomly chosen recipients aged 60 years in the cohort, with variations only in the Kidney Donor Risk Index (KDRI).

Supplementary Figure 2 Random survival forest-generated overall graft survival estimates for 4 randomly chosen recipients aged 70 years in the cohort, with variations only in the Kidney Donor Risk Index (KDRI).

Supplementary Figure 3 Random survival forest-generated patient survival estimates for 2 randomly chosen recipients aged 60 years in the cohort, with variations only in the Kidney Donor Risk Index (KDRI).

Supplementary Figure 4 Random survival forest-generated patient survival estimates for 2 randomly chosen recipients aged 70 years in the cohort,with variations only in the Kidney Donor Risk Index (KDRI).
