# Supplementary figures and images for: A novel machine learning approach generates personalized estimates of graft and patient survival to better inform older kidney transplant candidates
Source: Front Immunol. 2026 Jul 8;17:1881830. doi: 10.3389/fimmu.2026.1881830 (PMC13388154; doi:10.3389/fimmu.2026.1881830)

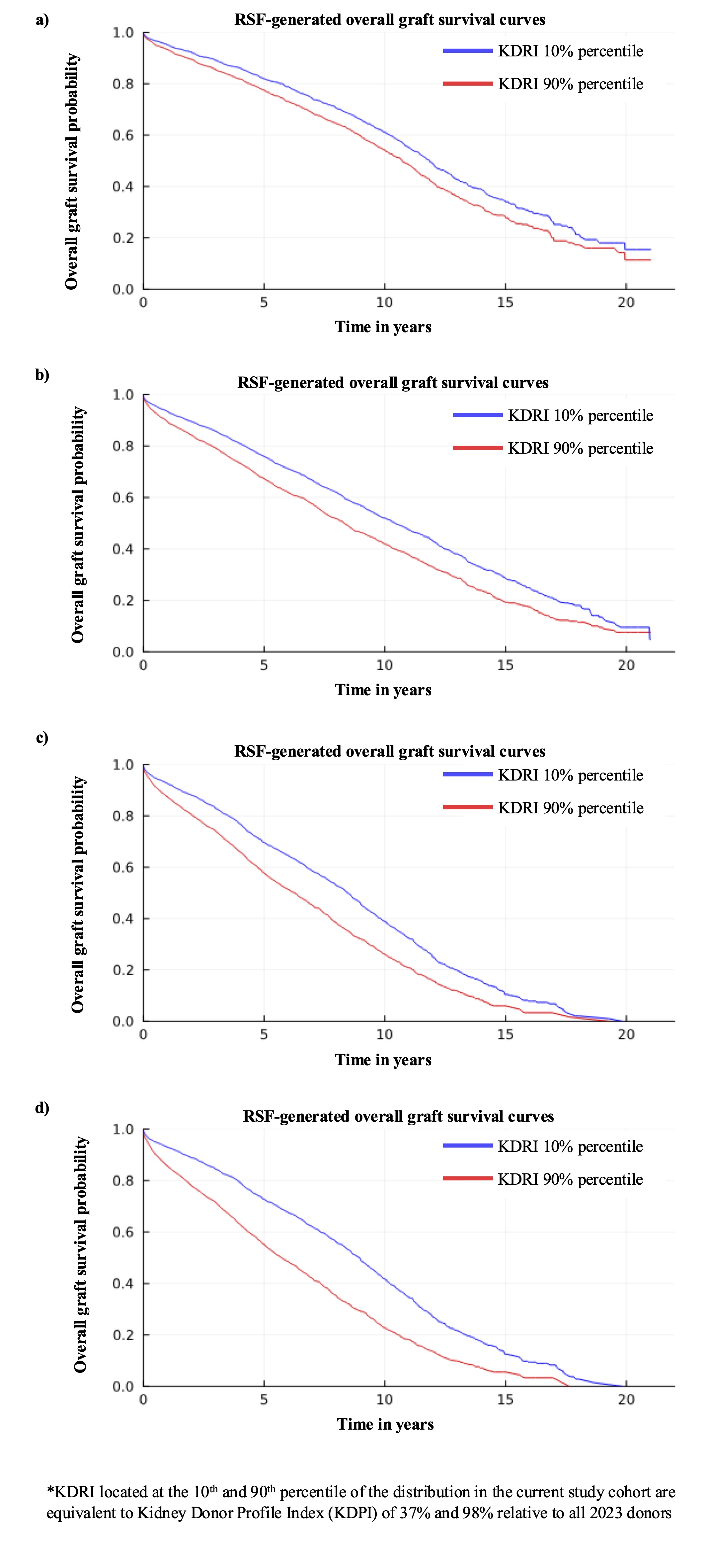

Supplement: Supplementary Figure 1 — Random survival forest-generated overall graft survival estimates for 4 randomly chosen recipients aged 60 years in the cohort, with variations only in the Kidney Donor Risk Index (KDRI). [file Image1.jpeg]

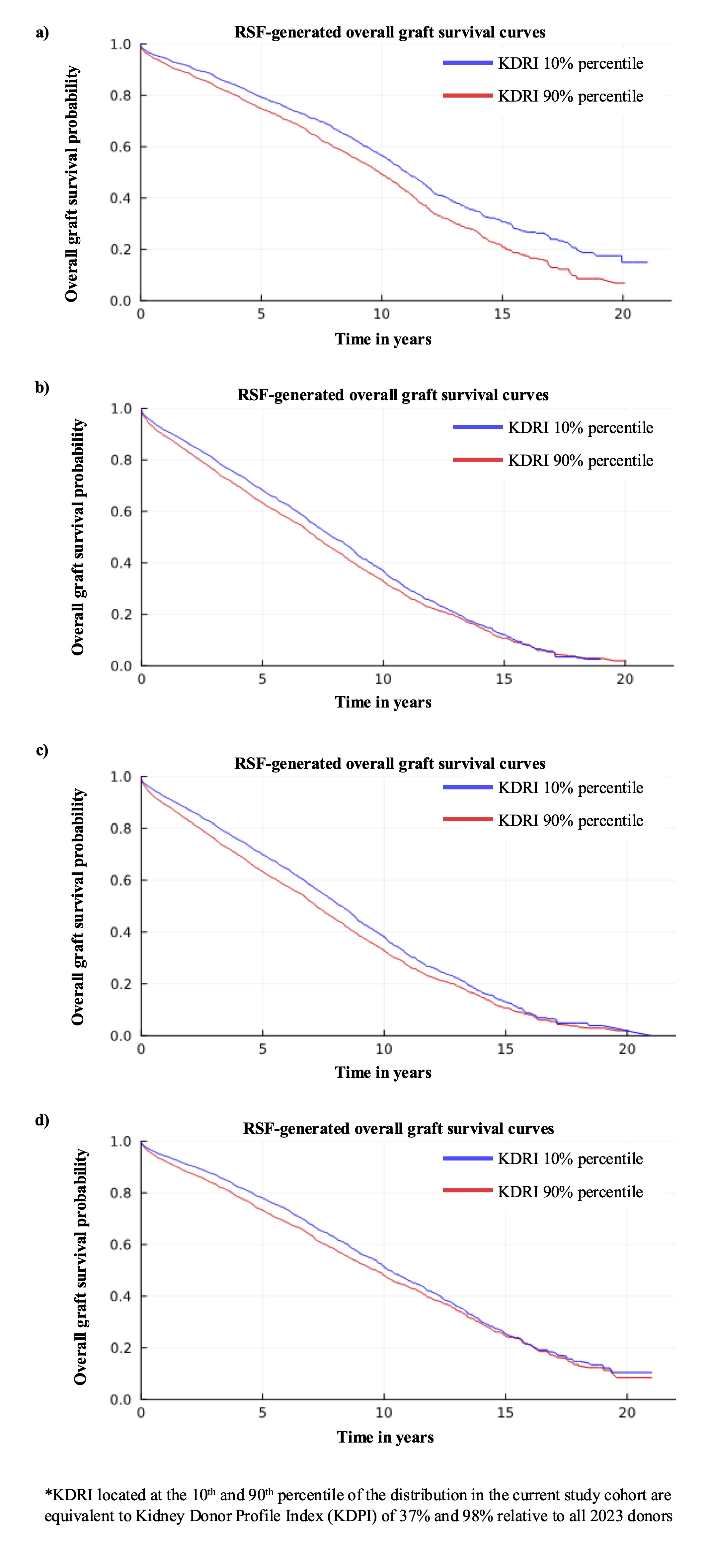

Supplement: Supplementary Figure 2 — Random survival forest-generated overall graft survival estimates for 4 randomly chosen recipients aged 70 years in the cohort, with variations only in the Kidney Donor Risk Index (KDRI). [file Image2.jpeg]

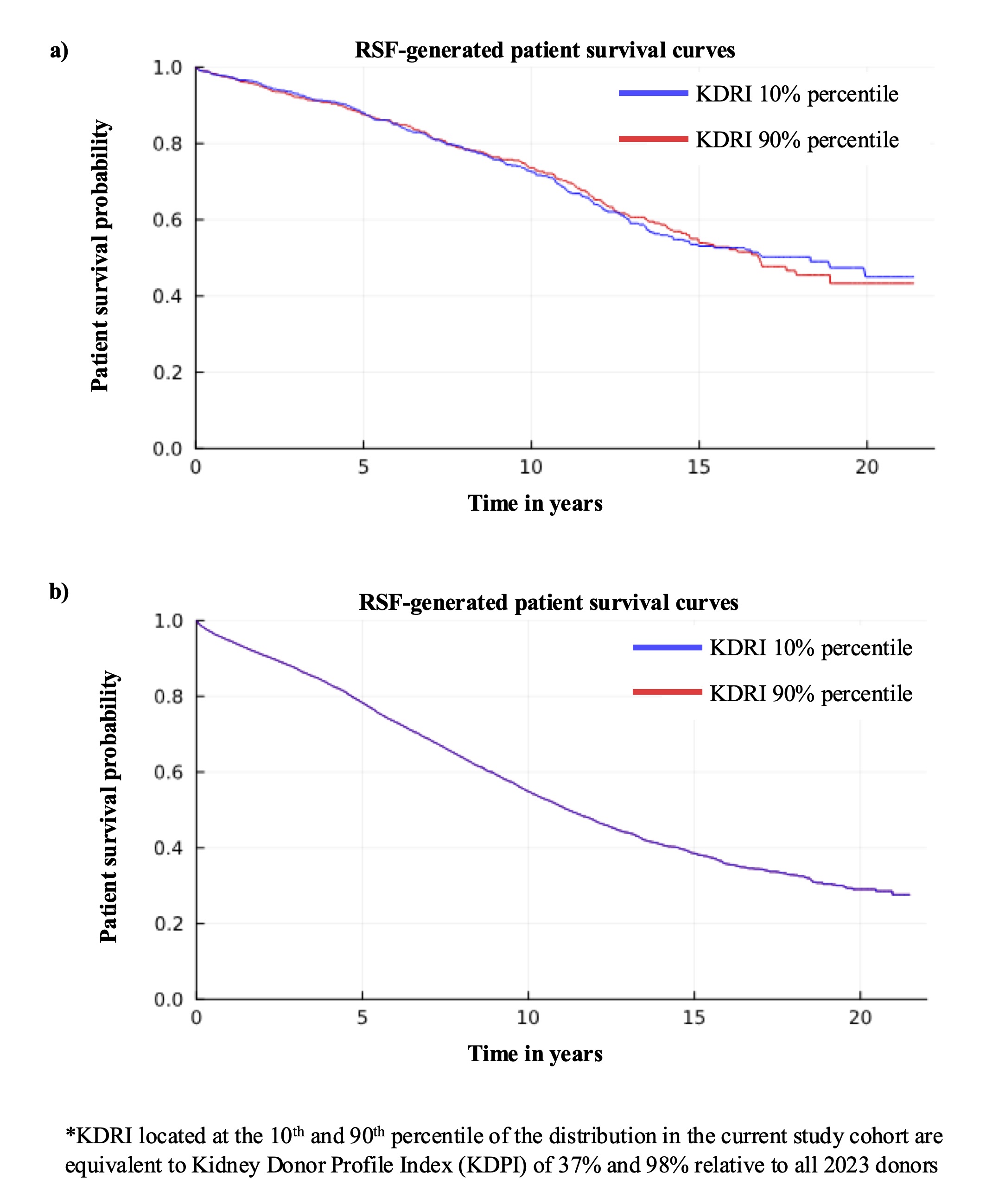

Supplement: Supplementary Figure 3 — Random survival forest-generated patient survival estimates for 2 randomly chosen recipients aged 60 years in the cohort, with variations only in the Kidney Donor Risk Index (KDRI). [file Image3.jpeg]

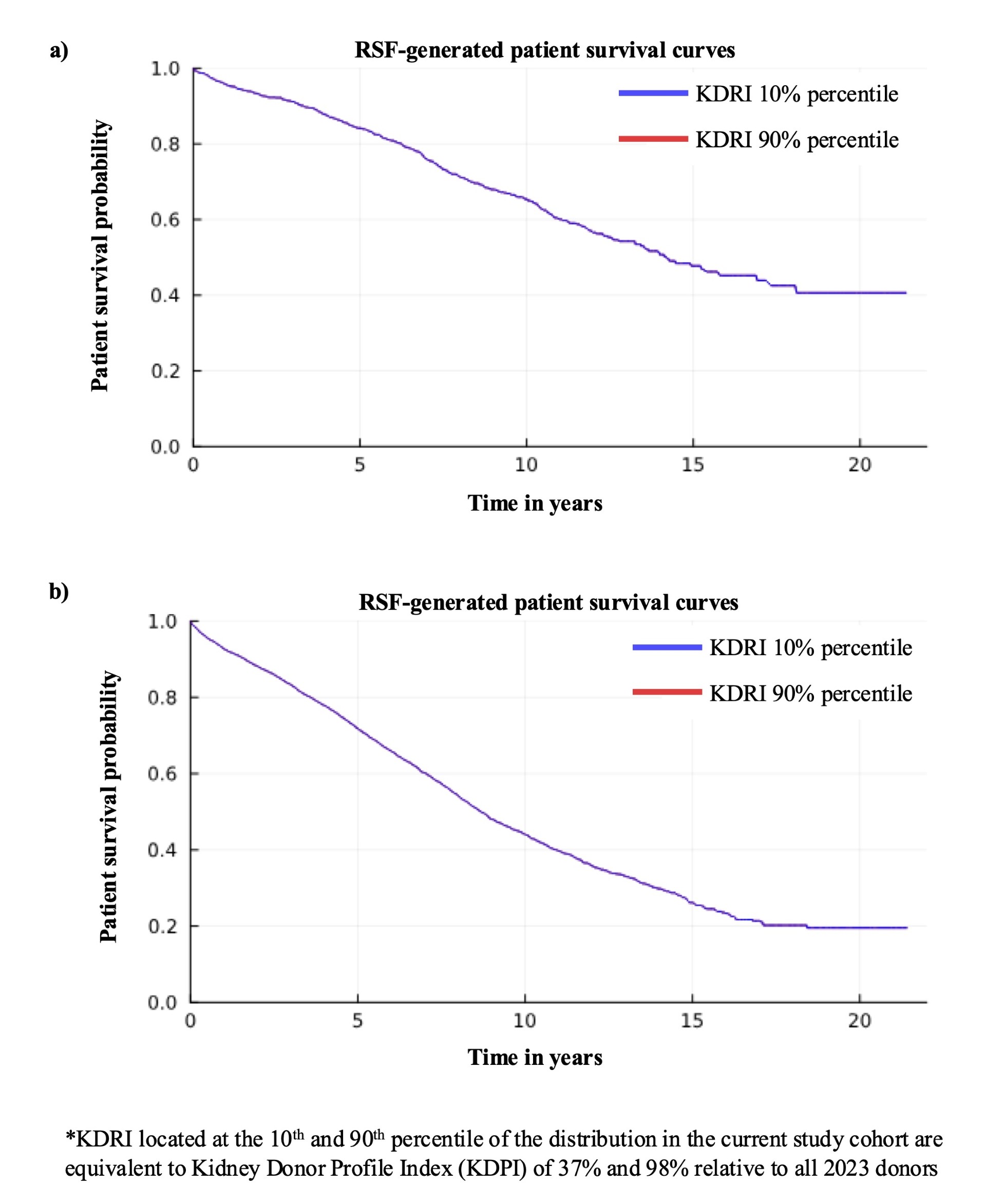

Supplement: Supplementary Figure 4 — Random survival forest-generated patient survival estimates for 2 randomly chosen recipients aged 70 years in the cohort,with variations only in the Kidney Donor Risk Index (KDRI). [file Image4.jpeg]
